# CDK12 Inactivation Attenuates Prostate Cancer Progression by Inhibiting BNIP3‐Mediated Mitophagy

**DOI:** 10.1111/cpr.70091

**Published:** 2025-07-02

**Authors:** Mengjun Huang, Hanqi Lei, Tongyu Tong, Hailin Zou, Binyuan Yan, Fei Cao, Yiting Wang, Qiliang Teng, Bin Xu, Juan Luo, Yupeng Guan, Shaohong Lai, Peng Li, Jun Pang

**Affiliations:** ^1^ Department of Urology, Pelvic Floor Disorders Center The Seventh Affiliated Hospital, Sun Yat‐sen University Shenzhen China; ^2^ Scientific Research Center The Seventh Affiliated Hospital, Sun Yat‐sen University Shenzhen China

**Keywords:** BNIP3, CDK12, enzalutamide treatment, mitophagy, prostate cancer

## Abstract

Mitochondrial stress‐induced mitophagy plays a critical role to maintain cellular homeostasis; however, in cancer cells, this process may also contribute to drug resistance. Our previous work identified CDK12 as a critical regulator of prostate cancer (PCa) cell survival under sustained enzalutamide exposure, though the precise mechanism remains to be elucidated. In this study, we hypothesize that CDK12 plays a key role in mitophagy regulation under mitochondrial stress, potentially modulating PCa cell resistance to enzalutamide, the first‐line clinical medication in PCa therapy. Utilising multiple in vitro PCa cell models, we demonstrate that both CDK12 knockdown and pharmacological inhibition with THZ531 impaired mitophagy following treatment with enzalutamide and mitophagy inducer CCCP. Mechanistically, our finding reveal that CDK12 inhibition disrupts FOXO3‐induced BNIP3 transcription, thereby preventing receptor‐mediated mitophagy and sensitising PCa cells to enzalutamide. This study identifies the CDK12‐FOXO3‐BNIP3 pathway as a novel regulatory mechanism governing mitophagy under mitochondrial stress. Importantly, these results underscore CDK12's role in preserving mitochondrial function and promoting PCa cell survival during enzalutamide treatment. These findings highlight the therapeutic potential of targeting the CDK12‐BNIP3‐mitophagy axis in combination with antiandrogen therapies, offering a promising strategy to overcome drug resistance in PCa and improve clinical outcomes.

## Graphical abstract

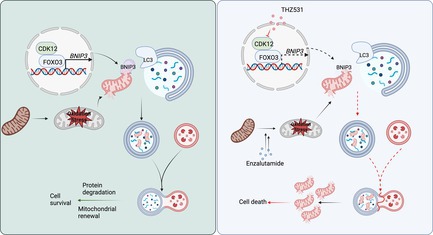

## 1 Introduction

The treatment of advanced prostate cancer (PCa) primarily targets the androgen receptor (AR) signalling pathway, employing androgen receptor pathway inhibitors (ARPIs) and androgen deprivation therapy (ADT). Although initially effective, these therapies often lead to disease recurrence as castration‐resistant prostate cancer (CRPC) emerges [1, 2]. Drug resistance arises through a diverse array of mechanisms, including overexpression and gene amplification of AR [3], altered expression of AR coactivators and core inhibitors [4] and the generation of AR splice variants such as AR‐V7 [5]. Additionally, alternative survival and growth pathways independent of AR activation, as well as metabolic and lineage plasticity under chronic therapeutic stress, contribute to drug resistance in CRPC patients [6, 7, 8]. Despite these insights, the mechanisms underlying PCa's response to ARPIs/ADT remain incompletely incompletely elucidated.

Mitophagy, a selective autophagy process, is crucial for degrading damaged mitochondria, thereby mitigating reactive oxygen species (ROS) accumulation and preserving cellular function [9, 10, 11]. It is primarily regulated by two pathways: the PINK1‐PRKN‐dependent pathway and receptor‐mediated pathways, such as those involving BCL2/adenovirus E1B 19 kDa interacting protein 3 (BNIP3) and BNIP3‐like (BNIP3L/NIX) [9, 12, 13, 14]. Recent research has revealed a significant role for mitophagy/autophagy in the progression of PCa into CRPC. For instance, enzalutamide treatment has been demonstrated in earlier research to be able to cause autophagy and mitophagy in PCa cells. Through this self‐defence mechanism, PCa cells develop resistance to enzalutamide and other ADTs, offering novel insights and strategies for advanced PCa treatment [15, 16, 17]. Nevertheless, the underlying mechanism in this process has yet to be thoroughly clarified.

Cyclin‐dependent kinase 12 (CDK12) is a key regulator of transcription elongation, DNA damage response and genomic stability [18, 19, 20, 21, 22]. Emerging evidence links CDK12 to tumour progression, with its inhibition potentially enhancing sensitivity to certain therapies [6, 23, 24, 25]. Additionally, recent studies indicate that inhibiting CDK12 increases oxidative stress in PCa cells, and suppressing CDK12 may trigger autophagy [26, 27]. However, the specific mechanisms connecting CDK12 to mitophagy are still not well understood. To this end, this study aims to investigate the role of CDK12 in mitophagy regulation under mitochondrial stress.

In this study, we identified a novel regulatory pathway that CDK12 was involved in the induction of mitophagy in response to mitochondrial stress. Specifically, CDK12 can promote nuclear localization of FOXO3, which in turn induces the BNIP3 gene transcription. Loss of CDK12 function using either small molecule inhibitor (THZ531) or RNA interference technology in PCa cells prevents the initiation of receptor‐mediated mitophagy by disrupting the FOXO3‐BNIP3 axis. More importantly, combination of CDK12 inhibition and enzalutamide treatment significantly suppresses tumour progression in a xenograft model. Overall, our findings not only reveal a new regulatory mechanism for CDK12‐FOXO3‐BNIP3‐mediated mitophagy, but also provide translational implications of targeting CDK12 for sensitising PCa treatment by ARPIs.

## 2 Materials and Methods

### 2.1 Cell Culture and Reagents

In this study, the LNCaP, C4–2B and RM‐1 cell lines were cultured in RPMI1640 medium (Gibco), and HEK293T cells were in high‐glucose DMEM (Gibco), which was enriched with 10% fetal bovine serum (FBS) from Bio‐Channel and 1% penicillin/streptomycin. All experiments were performed with mycoplasma‐free cells. The information regarding the chemical reagents used in this study is provided in Table S1.

### 2.2 Cell Transfection, Lentiviral Production and Infection Assays

The plasmids used for subcloning human CDK12, FOXO3 and mouse CDK12, which either overexpress or knockdown these genes, were obtained from DINGKE company. The sequence information of the shRNA is provided in Table S2. Cell transfection, lentiviral production and infection assays were conducted following previously established protocols [28]. In brief, the mixture(4 μg overexpression or shRNA plasmid + 3 μg the packaging plasmid pSPAX2 + 2 μg of the envelope‐expressing plasmid pMD2G) was transiently co‐transfected into HEK293T cells using Lipofectamine 2000 (Invitrogen). After 48 h, the lentivirus‐containing supernatant was collected and filtered. The PCa cells were subsequently transduced in the presence of 5 μg/mL polybrene for 24 h, followed by selection using 1 μg/mL puromycin for 48 h.

### 2.3 RNA Extraction, Reverse‐Transcription and Real‐Time PCR

In summary, the TRIzol extraction method was used for isolating RNA. 1 μg of total RNA was then used as a template for reverse transcription, utilising the Evo M‐MLV RT Reagent Kit (ACCURATE BIOLOGY) in accordance with the provided guidelines. Real‐time PCR was performed with the SYBR Green Pro Taq HS Reagent Kit (ACCURATE BIOLOGY) on a Bio‐Rad CFX96 Real‐Time Thermocycler (CFX96, Bio‐Rad Laboratories, Hercules, CA). The expression level of ACTIN served as an internal control. Sequence information for primers used for qPCR is provided in Table S3.

### 2.4 Western Blotting

The PCa cells were lysed using the protein lysis buffer (inhibitors of proteases and phosphatases) and incubated for 30 min on ice. The cell lysates were then resolved on 10% SDS‐PAGE and electro‐transferred to PVDF membranes (Millipore). The membranes were blocked with 5% nonfat dry milk and incubated with primary antibody overnight at 4°C. Detailed information on the primary antibodies is provided in Table S4.

### 2.5 Co‐Immunoprecipitation (co‐IP)

In brief, the human cells, including 293 T and PCa cells are lysed first with the protein lysis buffer, containing 50 mM Tris–HCl (pH = 7.5), 100 mM NaCl, 1% Triton X‐100, 0.1 mM EDTA, 0.5 mM MgCl2 and 10% glycerol. Then the cell lysates are incubated with antibody‐bound beads at 4°C overnight. Finally, the antibody/protein complexes are washed with lysis buffer for five times, boiled with protein loading buffer and subjected to western blotting analysis.

### 2.6 Mitochondrial Isolation

The Cell Mitochondria Isolation Kit (Beyotime, C3601) was utilised to isolate mitochondria from PCa cells. Following the separation of mitochondria and cytoplasm through differential centrifugation, the resulting fractions were preserved in a storage solution containing phenylmethylsulfonyl fluoride for subsequent western blotting analysis.

### 2.7 Analysis of Mitochondrial ROS

Mitochondrial ROS levels in living PCa cells were measured using the fluorescent indicator mitoSOX Red (HY‐D1055, MCE). The cells were incubated with mitoSOX at a concentration of 5 μM for 15 min at 37°C. The cells were then gently collected and analysed. The results are presented as a histogram illustrating the mean intensity of mitoSOX fluorescence.

### 2.8 Determination of Total Cellular ATP

The ATP Assay Kit (MA0440, MeilunBio) was then employed to prepare the standard curve, formulate the ATP detection working solution and measure ATP concentrations. The relative light units were determined using a luminometer.

### 2.9 The mtDNA Copy Number and Mitochondrial Mass Detection

To determine the mtDNA copy number, total DNA was extracted using the TIANamp Genomic DNA Kit (Tiangen Biotech). The mtDNA copy number was subsequently assessed through quantitative real‐time PCR, with genomic DNA serving as a loading control.

To detect total mitochondrial mass, cells were dissociated into single‐cell suspensions and then stained with 100 nM MitoTracker Green for 15 min at 37°C. Following staining, the cells were washed and analysed using a CytoFLEX LX flow cytometer (Beckman).

### 2.10 Luciferase Assay

Plasmids containing promoter of BNIP3 were designed by DINGKE (Shenzhen, China). They were co‐transfected into PCa cells with either NC or FOXO3 overexpression plasmid, respectively. After 48 h, cells were harvested, and the luciferase activity was examined using a Dual‐Luciferase Reporter Assay System (Promega) according to the manufacturer's instructions. Data were normalised to Renilla to control for transfection efficiency and presented as firefly/Renilla luciferase activity.

### 2.11 Immunofluorescence and Microscopy

Images were captured using a ZEISS LSM900 confocal microscope, and image analysis was conducted with ZEISS software, while co‐localization puncta quantification was carried out using ImageJ software.

### 2.12 Xenograft Model

All experimental protocols were reviewed and approved by the Animal Experimentation Ethics Committee of Sun Yat‐sen University. C4–2 cells (1 × 10^6^) were injected into the armpits of the Balb/c nude male mice (4 weeks old) and RM‐1 cells (2 × 10^5^) were injected into the armpits of the C57BL/6 male mice (4 weeks old). Once the average tumour volume reached 50–60 mm^3^, the mice were randomly assigned to one of four groups: the vehicle group, the Enzalutamide (Enza) group (30 mg/kg/day, prepared in a solution of DMSO, PEG300 and water at ratios of 5%:30%:65%, administered intraperitoneally [8]), the NP@THZ531 group (5 mg/kg, administered intraperitoneally once every 2 days [29]), and the Enza + NP@THZ531 group. The treatment continued for a total of 20 days. Tumour volumes were calculated using the formula (Volume = a × b^2^ × 0.5), where ‘a’ is the diameter perpendicular to ‘b’, which is the smallest diameter.

### 2.13 Statistical Analysis

Statistical analysis was conducted using GraphPad Prism 9.0 software. All experiments were performed a minimum of three times, and the results are presented as mean ± standard deviation where applicable. Statistical analysis was performed with the unpaired t test for two groups or one‐way ANOVA used for multiple groups, with a *p* < 0.05 considered statistically significant.

## 3 Results

### 3.1 CDK12 Is Required for the Proper Initiation of Mitophagy in Response to Mitochondrial Stress

**FIGURE 1:**
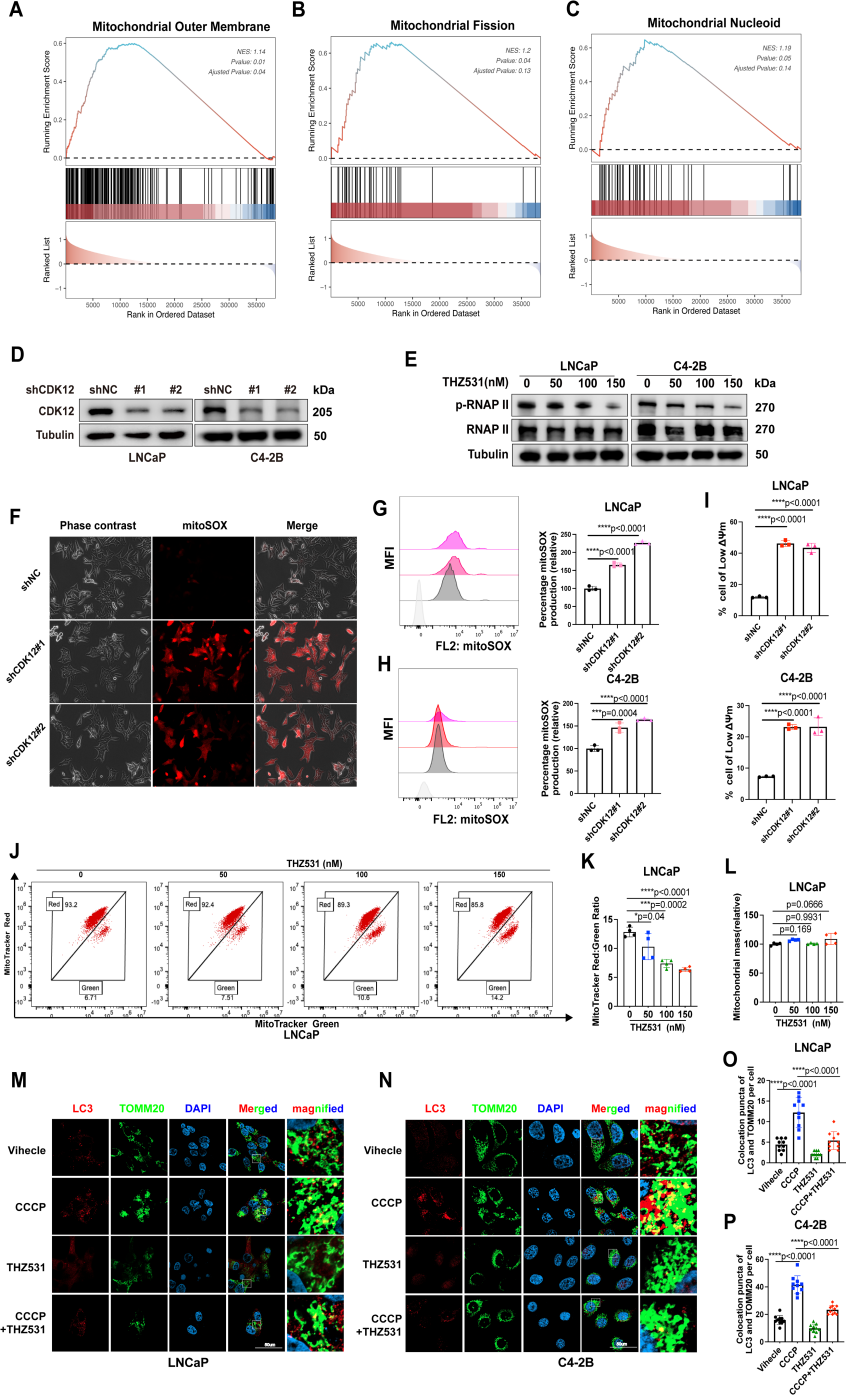
Inhibition of CDK12 induces mitochondrial dysfunction and suppresses mitophagy. (A–C) GSEA was used to analyse mitochondrial related signalling pathways regulated by CDK12 in PCa. (D) LNCaP and C4‐2B cells were transfected with shRNAs targeting different regions of the *CDK12* gene or the control vectors, and CDK12 expression was subsequently detected by western blot analysis. (E) PCa cells were treated with THZ531 at gradually increasing doses, and the levels of RNA polymerase II (RNAP II) and the phosphorylation of its CTD residues were analysed by western blotting to confirm the drug's inhibitory effect. (F–H) PCa cells stably expressing control or shCDK12 plasmid were incubated with mitoSOX probe, followed by fluorescence imaging analysis (F) and flow cytometry analysis (G and H) to quantify mitochondrial ROS levels. (I) Mitochondrial membrane potential was assessed by flow cytometry after incubation of PCa cells with the JC‐1 probe. (J–L) MitoTracker Red and MitoTracker Green staining, and quantification of mitochondrial mass in PCa cells were measured by flow cytometry. The ratio of red to green fluorescence intensity, which indicated the number of heathy mitochondria, was quantified. (M and N) LC3 and mitochondria (marked by TOMM20) were visualised by confocal microscopy. (O and P) Quantification of tumour cells containing colocalization puncta among LC3 and TOMM20 per cell was performed (*n* = 10, one‐way ANOVA). Scale bars = 50 μm.

In order to clarify the specific mechanism of CDK12's involvement in mitochondrial function, we initially used the public database, The Cancer Genome Atlas Program (TCGA), to perform mitochondrial‐related gene enrichment analysis. The results revealed significant upregulation of genes associated with the mitochondrial outer membrane protein pathway, mitochondrial fission pathway and mitochondrial nucleoid pathway (Figure 1A–C). We then assessed the expression levels of CDK12 across various cell lines. Given that LNCaP (AR‐positive, androgen‐dependent) and C4‐2B (AR‐positive, androgen‐independent) exhibited robust CDK12 expression (Figure S1A), these cell lines were selected for subsequent loss‐of‐function studies. To investigate the relationship between CDK12 and mitochondrial homeostasis, we measured the level of mitochondrial ROS (mitoSOX), a critical indicator of mitochondrial activity [30]. Indeed, pharmacological inhibition using THZ531 and gene knockdown of CDK12 led to an increase in mitochondrial ROS generation (Figure 1D–H, Figure S1B–E). A decrease in mitochondrial membrane potential (MMP) also confirmed mitochondrial damage (Figure 1I, Figure S1F,G). Meantime, THZ531 treatment decreased the staining ratio of MitoTracker Red to MitoTracker Green and exacerbated the accumulation of damaged mitochondria with a dose‐dependent manner (Figure 1J–L, Figure S1H–J). When treated with the mitophagy inducer CCCP, a reduction in genomic mtDNA was observed; however, the combination of CCCP and THZ531 mitigated this reduction (Figure S1K). These results collectively indicate that CDK12 inhibition leads to mitochondrial damage and impairs mitochondrial degradation, highlighting the critical role of CDK12 in maintaining mitochondrial integrity.

Malfunctioning mitochondria typically undergo mitophagy to promote mitochondrial turnover and maintain mitochondrial homeostasis. Based on this, we hypothesised that CDK12 may play a role in regulating mitophagy. Confocal fluorescence imaging demonstrated that CCCP induced the aggregation of LC3 protein around mitochondria, which was significantly suppressed by THZ531 treatment (Figure 1M–P). Furthermore, we isolated mitochondrial components and assessed LC3 aggregation around mitochondria. The results revealed that CCCP significantly increased the level of LC3‐II in the mitochondrial fractions, while THZ531 treatment attenuated the CCCP‐induced increase in LC3‐II levels (Figure S1L,M). These findings collectively suggest that CDK12 is essential for the induction of mitophagy in response to mitochondrial stress.

### 3.2 CDK12 Regulates Mitophagy Through BNIP3‐Dependent Pathway

**FIGURE 2:**
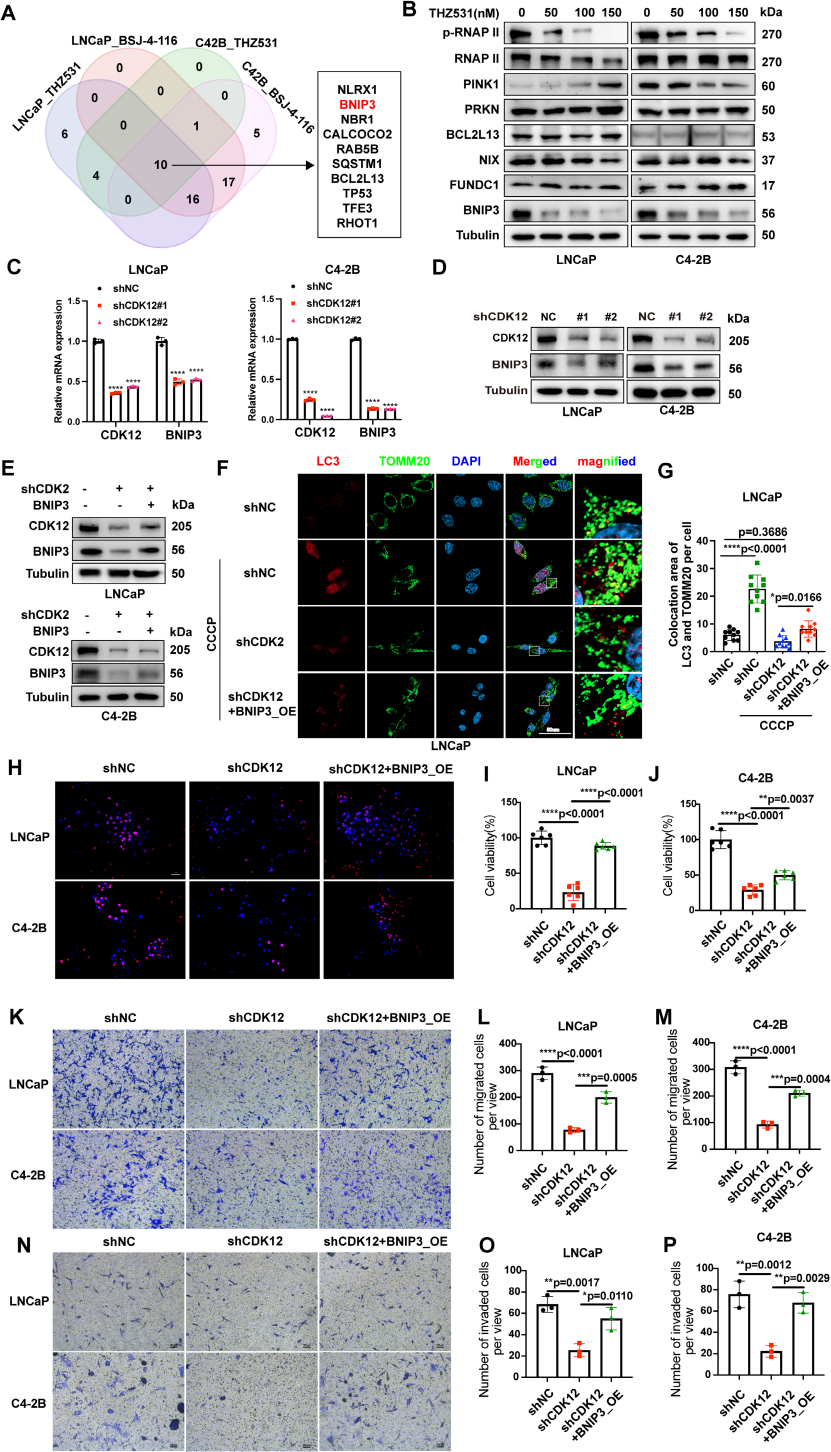
CDK12 modulates mitophagy induction upon mitochondrial stress through BNIP3. (A) Venn diagram was generated to illustrate the overlap of significantly differentially expressed mitophagy‐related genes across the groups. (B) PCa cells were treated with THZ531 at an indicated concentration for 24 h. Protein expression of PINK1, PRKN, NIX, BCL2L13, FUNDC1, BNIP3 were determined by western blotting. (C and D) qPCR and western blotting were performed to evaluate the expression of CDK12, BNIP3, and Tubulin (loading control) in PCa cells stably expressing shCDK12 plasmids. (E) Western blotting was conducted to determine the expression of CDK12, BNIP3 and Tubulin (loading control) in PCa cells stably expressing shNC, shCDK12 and shCDK12‐BNIP3_OE plasmids. (F and G) Confocal microscopy images were acquired to analyse the co‐localization of LC3 and TOMM20 in PCa cells stably expressing shNC, shCDK12 and shCDK12‐BNIP3_OE plasmids after treatment with CCCP. The number of co‐localization puncta between TOMM20 and LC3 per cell was quantified (*n* = 10, one‐way ANOVA). Scale bars = 50 μm. (H–P) Cell proliferation (H–J), migration ability (K–M), and invasion ability (N–P) were assessed in PCa cells stably expressing shCtrl, shCDK12 and shCDK12‐BNIP3_OE plasmids.

To further investigate the mechanism for CDK12‐mediated mitophagy, we conducted RNA‐seq analysis using PCa cells treated with THZ531 (a CDK12 kinase inhibitor) and BSJ‐4‐116 (a CDK12 protein degrader). The results revealed that the transcriptional level of *BNIP3* was significantly suppressed following either CDK12 kinase inhibition or CDK12 degradation (Figure 2A). Indeed, the subsequent qPCR and western blotting results also confirmed that either pharmacological inhibition or knockdown of CDK12 significantly downregulated BNIP3 expression, but hardly affected the PINK1 and PRKN expression levels (Figure 2B–D).

To investigate the role of the CDK12‐BNIP3 axis in mitophagy under cellular stress, we established PCa stable cell lines with CDK12 knockdown and rescued BNIP3 expression (Figure 2E). The knockdown of CDK12 did not lead to a significant reduction in the basal level of mitophagy in PCa cells (Figure 2F,G). However, upon treatment with CCCP, CDK12 knockdown significantly suppressed the co‐localization of mitochondria with LC3, indicating a reduction in mitophagy activity. Importantly, overexpression of BNIP3 in CDK12 knockdown cells alleviated the inhibitory effects on CCCP‐induced mitophagy (Figure 2F,G). These findings demonstrate that CDK12 regulates the initiation of stress‐induced mitophagy by modulating BNIP3 expression, thereby highlighting the critical role of the CDK12‐BNIP3 axis in this process.

In order to explore the functional impact of the CDK12‐BNIP3‐mitophagy axis on PCa cells, we assessed the proliferation, migration and invasion capabilities of the PCa stable cell lines. The results demonstrated that CDK12 knockdown significantly inhibited cell proliferation, which was partially restored upon BNIP3 reintroduction (Figure 2H–J). Similarly, migration and invasion assays revealed that CDK12 knockdown significantly suppressed these capabilities, and BNIP3 reintroduction partially restored the migratory and invasive capacities of CDK12 knockdown cells (Figure 2K–P). Notably, the restoration of BNIP3 did not fully recover the mitophagy flux suppressed by CDK12 knockdown, suggesting that BNIP3 is only a partial effector protein regulated by CDK12 in the context of mitophagy. Given the complex mechanisms underlying CDK12‐regulated mitophagy, further investigation into this process is warranted. Collectively, these findings highlight the role of CDK12 in receptor‐mediated mitophagy through BNIP3 regulation.

### 3.3 CDK12 Regulates the Expression of BNIP3 in a Manner Dependent on FOXO3 Transcriptional Activity

**FIGURE 3:**
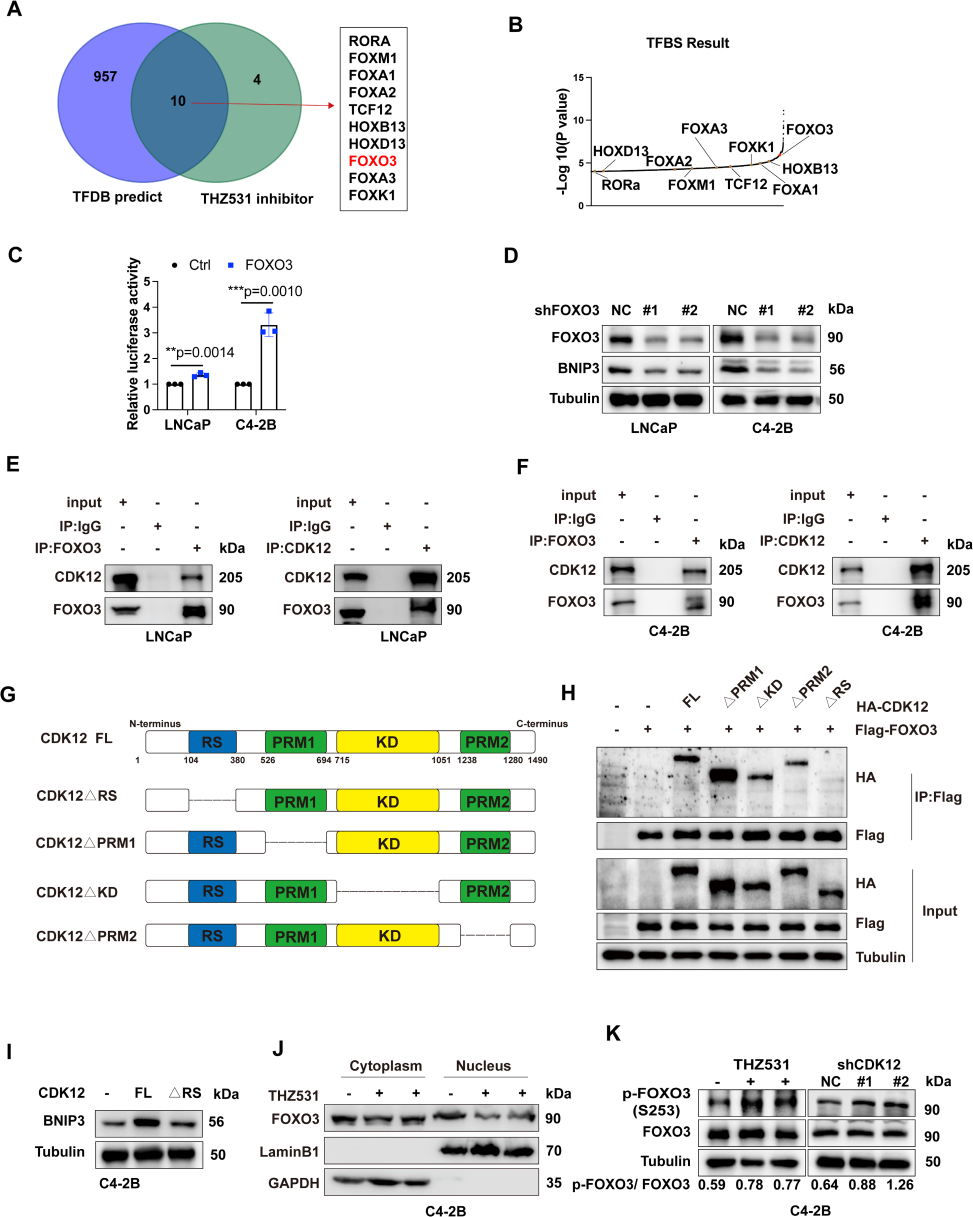
CDK12 regulates BNIP3 expression by interacting with FOXO3. (A) Venn diagram showing the intersection of TFs predicted for BNIP3 by AnimalTFDB database and the TFs with downregulated transcriptional activity after treatment with THZ531. (B) The significance scores of the 10 TFs screened from (A) in the AnimalTFDB database. (C) Luciferase activity was measured in PCa cells transfected with control or FOXO3 plasmids. (D) Protein levels of FOXO3, BNIP3, and Tubulin (loading control) were analysed in lysates of PCa cells stably expressing control or shFOXO3 plasmids. (E and F) Co‐IP analysis was performed to confirm the interaction between CDK12 and FOXO3 in LNCaP (E) and C4‐2B (F) cells. (G) Schematic diagram of indicated CDK12 truncation constructs. (H) Western blotting was conducted on input and co‐IP samples from HEK293T cells transfected with the indicated plasmids. (I) Western blotting was performed to assess BNIP3 protein expression in PCa cells transfected with control, CDK12 full‐length or RS domain‐truncated CDK12 plasmids. (J) Protein levels of FOXO3 in the cytoplasm and nucleus were analysed after treatment with 100 nM THZ531. (K) Western blotting was used to determine the levels of phosphorylated and total FOXO3 in PCa cells treated with 100 nM THZ531 or stably expressing shCDK12 plasmids.

To elucidate the regulatory mechanism of CDK12 on BNIP3, we queried the AnimalTFDB database and intersected the results with transcription factors (TFs) exhibiting significantly reduced activity in CUT&Tag assays [31]. This analysis identified 10 potential TFs of BNIP3, with FOXO3 emerging as a candidate TF (Figure 3A,B, Figure S2A,B). Notably, previous studies have implicated FOXO3 in the regulation of mitochondrial function through transcriptional upregulation of BNIP3 [32]. Luciferase assays confirmed that FOXO3 binds to the BNIP3 promoter (Figure 3C). Consistently, FOXO3 knockdown in PCa cells led to a significant decrease in both BNIP3 mRNA and protein levels (Figure 3D, Figure S2C).

We then investigated the interaction between CDK12 and FOXO3 proteins. Reciprocal co‐IP assays demonstrated a robust interaction between exogenous CDK12 and FOXO3 proteins in HEK293T cells, as well as endogenous interactions in PCa cells (Figure 3E,F, Figure S2D). To identify the specific domains of CDK12 responsible for binding to FOXO3, we generated three truncation mutants of CDK12 with an HA tag (Figure 3G). Co‐IP assays revealed that the arginine‐serine (RS)‐rich domain of CDK12 is essential for its interaction with FOXO3 (Figure 3H). Subsequent western blot analysis confirmed that overexpression of CDK12 lacking the RS domain failed to induce BNIP3 expression, unlike full‐length CDK12 (Figure 3I).

The nuclear localization of FOXO3 is closely associated with its transcriptional activity [33]. Nucleocytoplasmic separation experiments and western blot analysis indeed confirmed that CDK12 knockdown or pharmacological inhibition reduced the nuclear localization of FOXO3 (Figure 3J, Figure S2E). Interestingly, we observed that the phosphorylation of FOXO3 at serine 253, which promotes its cytoplasmic retention, was dramatically increased following CDK12 knockdown or inhibition (Figure 3K). Collectively, these findings suggest that CDK12 regulates the transcriptional level of BNIP3 by modulating FOXO3 activity.

### 3.4 CDK12 Deficiency Enhanced Mitochondrial ROS Production But Prevented Mitophagy in Enzalutamide‐Treated PCa Cells

**FIGURE 4:**
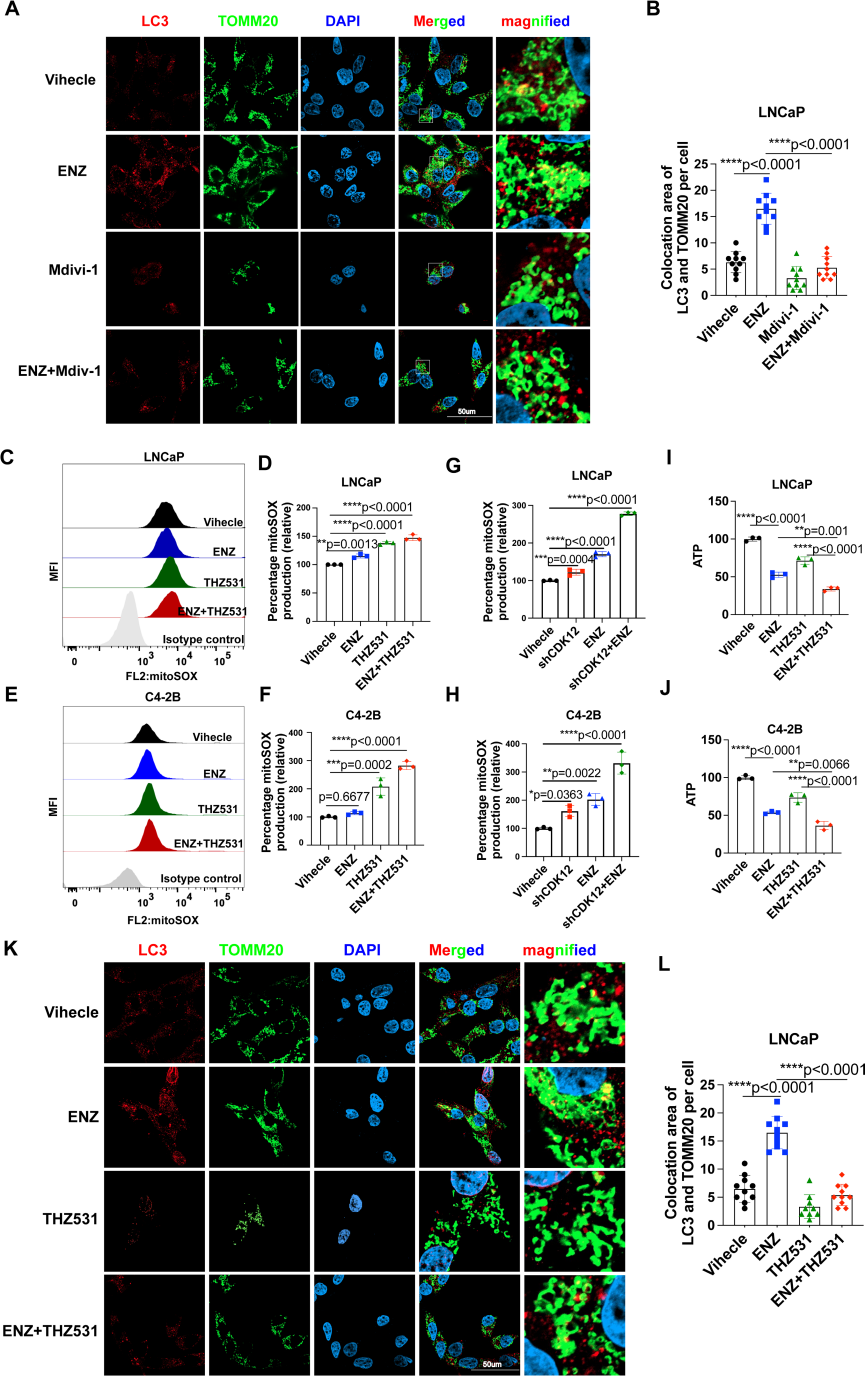
Inhibition of CDK12 exacerbates enzalutamide‐induced ROS stress while blocking mitophagy. (A and B) LC3 and mitochondria (marked by TOMM20) were visualised by confocal microscopy after treatment with enzalutamide, Mdivi‐1 or their combination. The number of colocalization puncta between TOMM20 and LC3 per cell was quantified (*n* = 10, one‐way ANOVA). Scale bars = 50 μm. (C–F) PCa cells were treated with THZ531, enzalutamide or both for 24 h, followed by incubation with the mitoSOX probe. Mitochondrial ROS levels were subsequently measured by flow cytometry. (G and H) Mitochondrial ROS levels were measured by flow cytometry in shCDK12‐expressing PCa cells treated with or without enzalutamide for 24 h. (I and J) Total ATP production in PCa cells was measured in the presence of THZ531, enzalutamide or their combination for 24 h. (K and L) LC3 and mitochondria (marked by TOMM20) were visualised by confocal microscopy after treatment with enzalutamide, THZ531 or their combination. The number of colocalization puncta between TOMM20 and LC3 per cell was quantified (*n* = 10, one‐way ANOVA). Scale bars = 50 μm.

Previous studies have demonstrated that enzalutamide induces mitophagy in PCa cells [15, 16, 17]. Consistent with these findings, our experiments revealed a significant increase in LC3 aggregation around mitochondria following enzalutamide treatment (Figure 4A,B). Importantly, the mitophagy inhibitor Mdivi‐1 effectively suppressed enzalutamide‐induced LC3 and mitochondrial aggregation (Figure 4A,B). Given the involvement of CDK12 in mitophagy, we hypothesized that CDK12 inhibition might block enzalutamide‐induced mitophagy, thereby exacerbating the accumulation of damaged mitochondria. To test this, we treated PCa cells with THZ531 in combination with enzalutamide and assessed mitochondrial function. Results demonstrated that combined treatment significantly increased mitochondrial ROS levels (Figure 4C–F). Similar findings were observed in CDK12 knockdown cell lines (Figure 4G,H). Furthermore, cellular ATP content was significantly reduced in the combined treatment compared to either THZ531 or enzalutamide alone (Figure 4I,J). Confocal fluorescence imaging also showed that enzalutamide induced the aggregation of the protein LC3 around the mitochondria, while THZ531 suppressed this enzalutamide‐induced aggregation (Figure 4K,L). These findings collectively indicate that CDK12 inhibition disrupts the mitophagy pathway under stress conditions, leading to exacerbated mitochondrial dysfunction in PCa cells.

### 3.5 CDK12 Inhibition Synergizes With Enzalutamide to Suppress the Growth of PCa Cells

**FIGURE 5:**
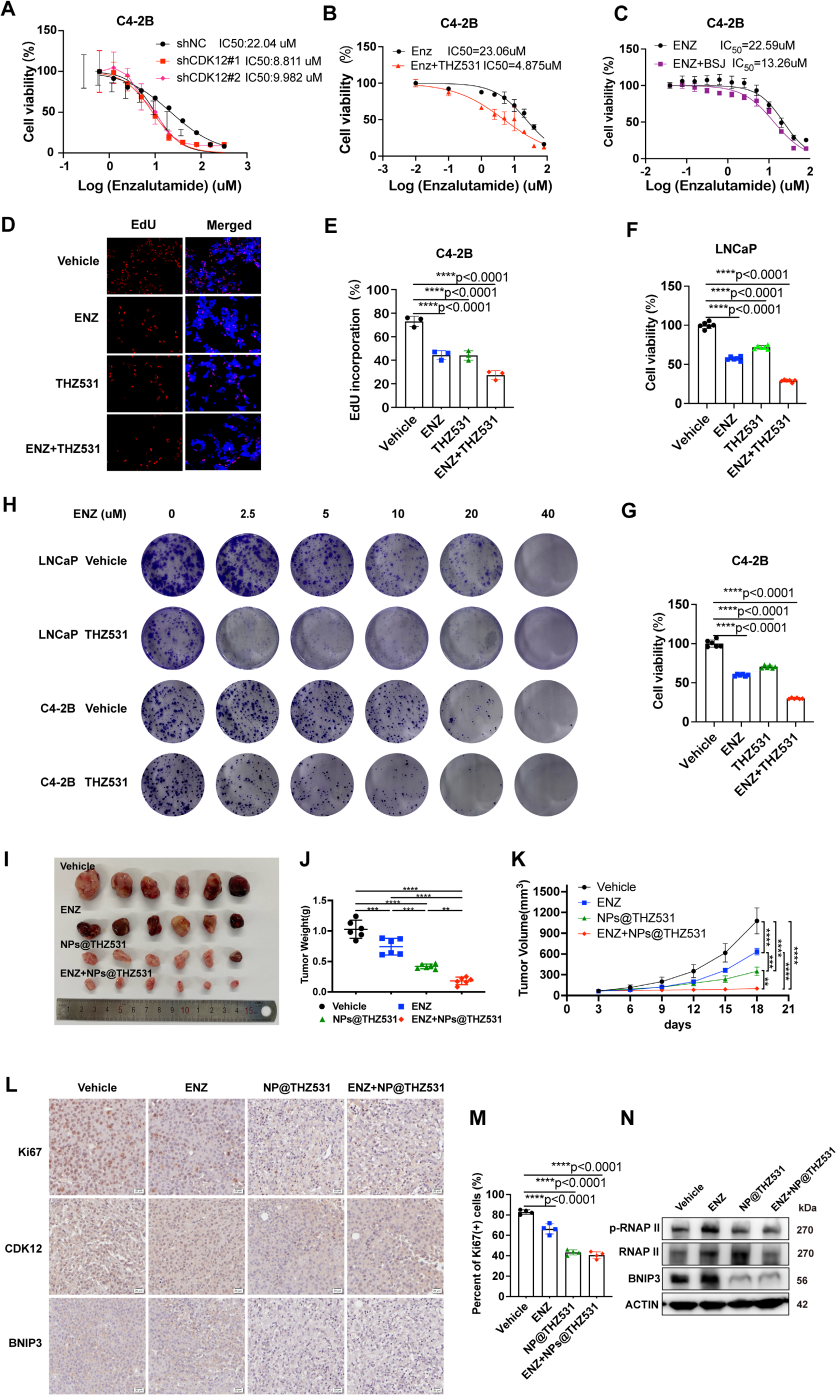
Synergistic effect of THZ531 combined with enzalutamide in vitro and in vivo. (A–C) Enzalutamide dose–response curves were generated in C4‐2B cells with (A) CDK12 knockdown, (B) CDK12 kinase inhibitor THZ531 or (C) CDK12 protein degrader BSJ‐4‐116. (D–H) CCK‐8, clone formation and EdU assays collectively demonstrated that THZ531 effectively inhibited the proliferation of PCa cells. (I–K) Xenograft tumour growth curve and the collected tumours derived from C4‐2B cells upon treatment with enzalutamide, NP@THZ531 alone or their combination. Tumour volume at various time points of treatment is measured using a vernier calliper. The quantitation data represent means ± SD, *n* = 6. (L and M) IHC analyses of Ki67, CDK12 and BNIP3 protein expression in C4‐2B cell‐derived xenograft tissues under treatment with enzalutamide, NP@THZ531 alone or their combination. Quantitation of Ki67+ cells represents means ± SD. Scale bar = 20 μm.(N)Protein expression of p‐RNAP II, RNAP II and BNIP3 was determined by western blotting.

To further investigate whether CDK12‐mediated mitophagy contributes to enzalutamide resistance in PCa cells, we conducted growth inhibition assays to assess drug sensitivity in PCa cell lines. The results demonstrated that PCa cell lines exhibited enhanced sensitivity to enzalutamide in the presence of CDK12 inactivation (Figure 5A–C, Figure S3A–D). Furthermore, cell viability analysis revealed that inhibiting CDK12 significantly increased the cytotoxic effects of enzalutamide on PCa cells (Figure 5D–H).

We then evaluated whether the combination of THZ531 and enzalutamide produces a synergistic effect in vivo. BALB/c nude mice bearing these C4‐2B tumours were then treated with enzalutamide (30 mg/kg, i.p., daily [8]) or NP@THZ531 (5 mg/kg, i.p., once every 2 days [29]) until the average volume of the tumour reached approximately 50–60 mm^3^. The combination of NP@THZ531 with enzalutamide significantly inhibited the growth of C4‐2B xenografts compared to treatment with the vehicle, NP@THZ531 or enzalutamide alone, as evidenced by measurements of tumour volumes and weights at the study's endpoint (Figure 5I–K). Similar results were observed in RM‐1 subcutaneous xenograft models using immunocompetent C57BL/6 mice (Figure S3E–H). IHC results revealed a significant reduction in tumour cell proliferation in vivo following the combination therapy (Figure 5L–N, Figure S3I–K). These findings collectively demonstrate that the combination of CDK12 inhibition and enzalutamide synergistically inhibits the growth of PCa xenograft tumours.

### 3.6 Clinical Validation of CDK12 and BNIP3 in PCa

**FIGURE 6:**
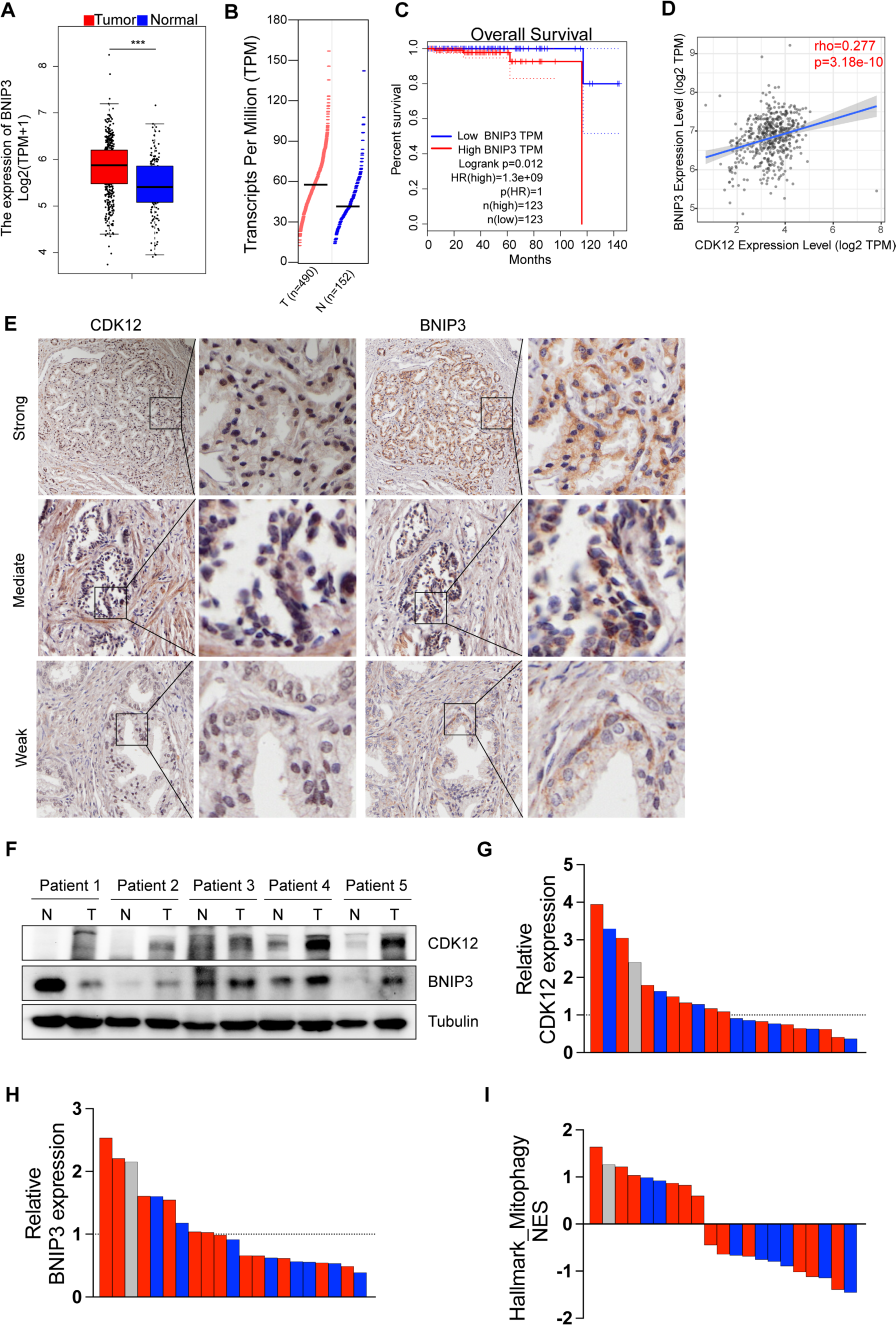
Clinical validation of CDK12 and BNIP3 in PCa. (A and B) Expression levels of BNIP3 mRNA in the TCGA dataset. (C) Kaplan–Meier analysis assessed the correlation between BNIP3 expression and overall survival in TCGA. (D) Correlation between CDK12 and BNIP3 in TCGA. (E) Representative staining with antibody against CDK12 and BNIP3 in PCa and paired nontumour tissues detected by IHC (scale bar: 100 μm). (F) Western blotting of proteins extracted from five paired samples of tumour (T) and paired nontumour tissues (N). (G–I) Expression levels of CDK12 and BNIP3, and GSEA of Hallmark_mitophagy in 21 mCRPC tumours before and after enzalutamide treatment from the Westbrook et al. [34] dataset.

We subsequently investigated the correlation between CDK12 and BNIP3 in clinical samples. First, we examined BNIP3 expression in PCa tissues leveraging the TCGA database, which revealed its increased expression in PCa tissues compared to normal tissues (Figure 6A,B). Kaplan–Meier analysis further highlighted that higher BNIP3 expression was associated with poorer overall survival in PCa patients(Figure 6C). Additionally, TIMER2.0 database analysis demonstrated a significant positive correlation between BNIP3 and CDK12 expression (Figure 6D). This correlation was further validated in PCa tissue samples, where protein expression of BNIP3 and CDK12 exhibited a positive association (Figure 6E). Furthermore, both CDK12 and BNIP3 expression levels were significantly higher in PCa tissues compared to adjacent normal tissues (Figure 6F).

In addition, to evaluate whether mitophagy‐associated resistance to AR blockade is evident in clinical samples, we analysed the RNA‐sequencing data from 21 mCRPC tumours before and after enzalutamide treatment in the Westbrook et al. dataset [34]. Our analysis revealed that CDK12 and BNIP3 expression levels in PCa tissues of most patients who did not respond to enzalutamide treatment were markedly elevated compared to pretreatment levels (Figure 6G,H). Moreover, nine patients exhibited significant enrichment of the Hallmark_mitophagy pathway following enzalutamide treatment. Among these nine, six patients were classified as nonresponders, and one had an unknown response to the drug (Figure 6I). Collectively, these findings provide evidence for the interplay between CDK12, BNIP3 and mitophagy in PCa progression and therapeutic resistance.

## 4 Discussion

The AR signalling pathway remains the primary target for therapeutic interventions in advanced PCa. While ADT and ARPIs demonstrate initial effectiveness, patients ultimately face relapse and progress to treatment‐resistant metastatic CRPC [1, 2]. To combat the issue of resistance to ARPIs, there is an increasing need to identify and co‐target the mechanisms that contribute to ARPI stress during treatment.

**FIGURE 7:**
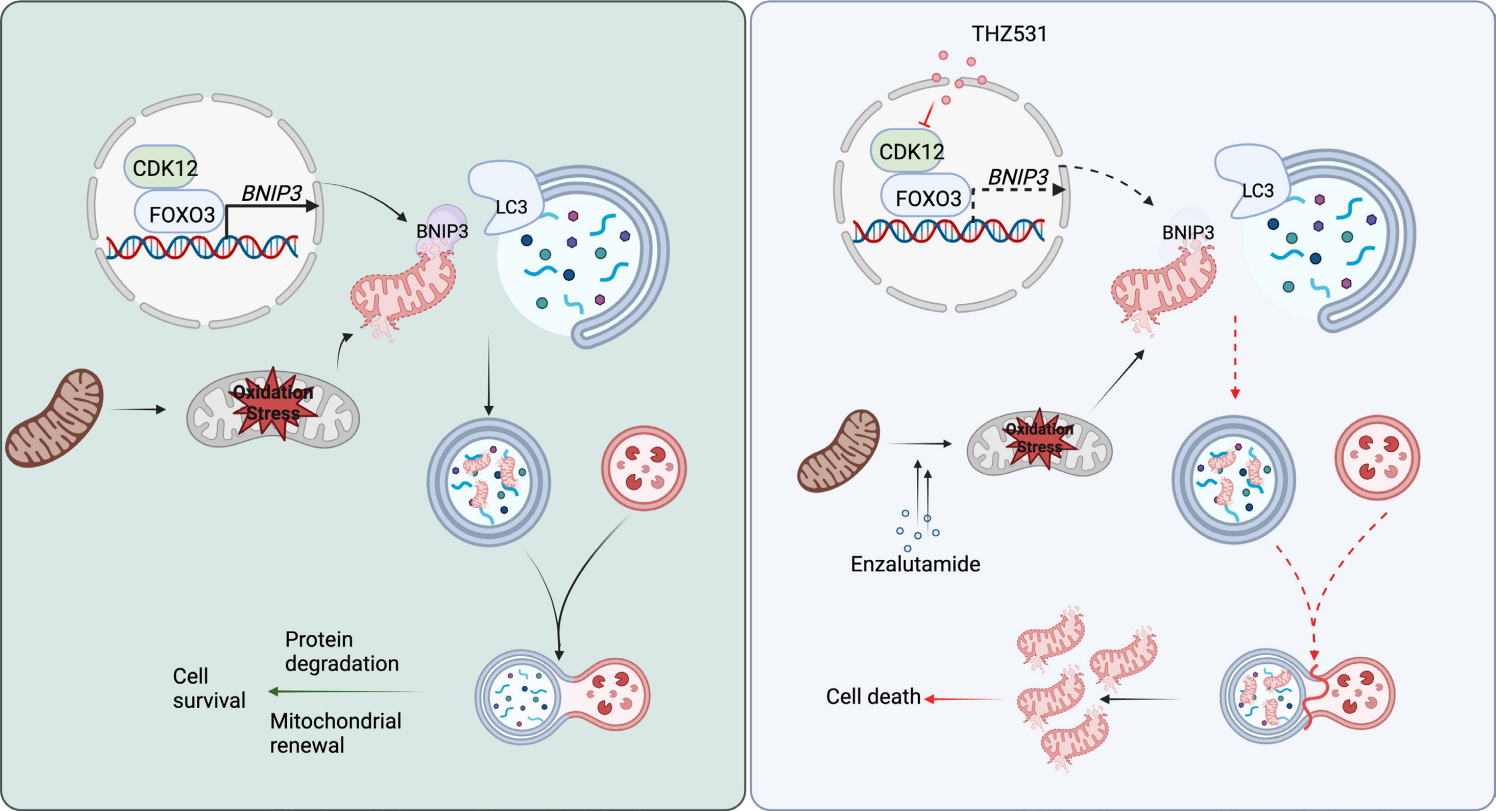
Schematic representation of THZ531 blockage of BNIP3‐mediated mitophagy and induction of mitochondrial dysfunction in PCa treatment. In response to enzalutamide, damaged mitochondria are targeted for mitophagic degradation through the binding of BNIP3 to LC3, forming autophagosomes that are subsequently transported to lysosomes for degradation. These self‐protective mechanisms promote mitochondrial renewal, reduce oxidative stress, and finally contribute to cancer cell survival. CDK12 facilitates the nuclear retention of FOXO3 through protein–protein interactions, which increases FOXO3‐regulated transcription of BNIP3. THZ531 suppresses CDK12, resulting in the downregulation of BNIP3 transcription and the inhibition of enzalutamide‐induced mitophagy, thereby enhancing the sensitivity of PCa to enzalutamide treatment.

Our previous research has identified CDK12 as a critical factor for the survival of PCa cells under sustained exposure to enzalutamide [31]. While earlier studies have predominantly focused on CDK12's role in DNA replication, transcription, splicing and DNA damage repair [21], recent findings suggest that CDK12 also plays a crucial role in tumour growth and progression. It functions as a super‐enhancer, collaborating with the relevant transcriptional complexes to facilitate the expression of downstream genes, thereby contributing to tumour progression and drug resistance [19, 31, 35, 36]. These findings also establish a new treatment paradigm that leverages the mechanism of tumour synthetic lethality [37, 38, 39, 40]. Nonetheless, there remains an absence of studies on how CDK12 maintains the homeostasis of PCa cells to survive and progress under stress. In this context, our finding indicated that CDK12 enhances cell viability by modulating autophagy and the process of mitophagy under various stress conditions. Inhibition of CDK12 significantly diminished mitophagy induction and exacerbated the reduction in ATP levels and cellular viability upon exposure to enzalutamide, highlighting the importance of CDK12 in maintaining mitochondrial homeostasis and promoting cellular survival under conditions of stress (Figure 7).

Our study presents evidence of the crucial function that CDK12 plays in promoting mitophagy under conditions of mitochondrial stress. In this study, we discovered that CDK12 is essential for the proper initiation of mitophagy in response to various mitochondrial stresses. Specifically, we observed that the induction of mitophagy following CCCP treatment was significantly reduced in cells where CDK12 was knocked down. Furthermore, the CDK12 inhibitor THZ531 also notably suppressed mitophagy induction in these cells when exposed to CCCP. Consistent with recent studies [26], we found that inhibition of CDK12 can induce excessive ROS production in cells, suggesting that CDK12 plays an important regulatory role in maintaining mitochondrial homeostasis. Mitophagy is regarded as a key protective mechanism that removes dysfunctional mitochondria, thereby maintaining mitochondrial ROS balance and improving overall mitochondrial quality [41, 42]. Our mechanistic analysis indicated that the mitophagy receptor BNIP3 plays a crucial role in the CDK12‐mediated induction of mitophagy. When CDK12 was inhibited, it hindered the initiation of receptor‐mediated mitophagy by disrupting the FOXO3‐BNIP3 signalling pathway and directly decreasing the transcription of BNIP3.

BNIP3‐dependent mitophagy is necessary for regulating ROS levels in developing tumours [43]. Tumour cells continue to generate ROS when subjected to stress, with mitochondria serving as the primary source of ROS production. However, if ROS are not effectively neutralised, they can lead to severe peroxidative damage within the cells. Recent studies have demonstrated that the heightened generation of superoxide resulting from damage to the mitochondrial electron transport chain acts as an alternative signal for the sequestration of mitochondria into autophagosomes [44, 45, 46]. In the present study, we utilised mitoSOX, a mitochondria‐targeted superoxide sensor, and observed that the inhibition of CDK12 led to an increase in mitochondrial ROS levels. This increase was further intensified by the simultaneous administration of enzalutamide. This suggests that the combination of CDK12 inhibition with enzalutamide significantly increases the levels of intracellular ROS. Simultaneously, this combination hinders the conversion of ROS through mitochondrial autophagy mediated by the CDK12‐BNIP3 pathway. As a result, ROS continue to accumulate, leading to irreversible damage to the cells.

Currently, mitophagy is a rapidly developing field. Emerging technologies, such as deep learning and artificial intelligence, can be utilised to design and screen drugs based on existing natural products and specific target proteins, thereby guiding the discovery and validation of new mitophagy regulators. The natural compound urolithin A has been shown to induce mitophagy both in vivo and in vitro [47, 48], and its clinical trials are currently ongoing (NCT05735886). Another natural polyphenolic compound, resveratrol, which regulates mitophagy, is also being tested in multiple clinical trials (NCT02123121, NCT04449198, NCT03728777, NCT02245932) [49]. However, most of these mitophagy‐inducing agents in clinical trials exhibit multifunctional properties, and their mechanisms remain unclear. Additionally, there is a lack of clinical trials directly targeting mitophagy. Understanding how to specifically target mitochondria to regulate mitophagy and how to specifically detect mitophagy flux could lead to breakthroughs in the clinical application of mitophagy‐targeted therapies. Our study has revealed the significant role of CDK12 in regulating mitophagy and ROS levels. Based on prior translational research findings [29], we have designed and synthesised small‐molecule compounds encapsulating THZ531 and conducted in vivo experiments to evaluate their potential as therapeutic targets, providing new approaches and insights for the treatment of PCa. Future studies will focus on the in vivo pharmacokinetics and tumour enrichment effects of CDK12 inhibitors.

This study also has certain limitations. First, our findings demonstrate that BNIP3 is a key downstream mediator of CDK12 during mitophagy under mitochondrial stress. However, the incomplete restoration of mitophagy flux under stress conditions in CDK12 knockdown cell lines upon BNIP3 supplementation suggests the potential existence of other regulatory mechanisms that warrant further investigation. Second, previous studies have identified BNIP3 as a critical receptor protein regulating mitophagy under hypoxic conditions. Given that the hypoxic tumour microenvironment in overgrown PCa tumours itself is a state of hypoxia, and enzalutamide treatment also induces hypoxic stress in PCa cells, it is essential to explore how BNIP3 regulates mitochondrial metabolism and energy supply under hypoxic conditions. Third, while this study highlights the preclinical significance of targeting CDK12 to enhance the therapeutic efficacy of enzalutamide in PCa by elucidating its regulatory role in mitophagy, the potential off‐target effects and toxicities of the CDK12 kinase inhibitor THZ531 and their associated side effects were not investigated. This aspect will inevitably require attention in future translational studies. Finally, although this study utilised both immunocompetent and immunodeficient mouse models to validate the synergistic effects of CDK12 gene knockdown and pharmacological inhibition, the models employed remain limited. Future studies should incorporate patient‐derived xenograft models and mouse orthotopic tumour models to more accurately simulate the tumour microenvironment of PCa patients.

In conclusion, we identified a novel regulatory pathway through which CDK12 is involved in the induction of mitophagy in response to mitochondrial stress. Mechanistically, CDK12 promotes FOXO3 localization to the nucleus, which subsequently induces the transcription of the BNIP3 gene. The loss of CDK12 function prevents the initiation of receptor‐mediated mitophagy by disrupting the FOXO3‐BNIP3 signalling axis. Notably, the combination of CDK12 inhibition and enzalutamide treatment significantly suppressed tumour progression in a xenografted PCa model. Overall, our findings suggest potential translational implications for targeting CDK12 to enhance the effectiveness of ARPIs in treating PCa.

## Author Contributions

**Mengjun Huang:** writing – original draft. **Hanqi Lei:** funding acquisition, writing – original draft. **Tongyu Tong:** supervision. **Hailin Zou:** methodology. **Binyuan Yan:** validation. **Fei Cao:** software. **Yiting Wang:** investigation. **Qiliang Teng:** validation. **Bin Xu:** project administration. **Juan Luo:** supervision. **Yupeng Guan:** funding acquisition. **Shaohong Lai:** validation. **Peng Li:** writing – review and editing. **Jun Pang:** funding acquisition, writing – review and editing.

## Ethics Statement

All experimental protocols were reviewed and approved by the Animal Experimentation Ethics Committee of Sun Yat‐sen University (SYSU‐IACUC‐2024‐001679). All human studies were approved by the Seventh Hospital of Sun Yat‐sen University Medical Ethics Committee (KY‐2024‐401‐01).

## Conflicts of Interest

The authors declare no conflicts of interest.

## Supporting information


**Figure S1.** Inhibition of CDK12 suppresses mitophagy.
(A) CDK12 protein expression levels in various prostate cell lines. (B–E) PCa cells treated with THZ531 at gradually increasing doses were incubated with mitoSOX probe, and then flow cytometry analysis were performed to detect mitochondrial ROS levels. (F,G) Mitochondrial membrane potential was analysed by flow cytometry after incubation of PCa cells with the JC‐1 probe. (H–J) MitoTracker Red and MitoTracker Green staining (I), and quantification of mitochondrial mass (J) in PCa cells were measured by flow cytometry. The ratio of red to green fluorescence intensity, which indicated the number of heathy mitochondria, was quantified. (K) mtDNA copy numbers of PCa cells were measured by quantitative PCR assay after treated with CCCP and/or THZ531. (L and M) C4‐2B cells were treated with 10 μM CCCP and/or 100 nM THZ531 for 24 h. The protein levels of LC3 in mitochondria and the cytoplasm were measured by western blotting. The protein level of the LC3‐II form in mitochondria was quantified and normalised to VDAC1.
**Figure S2**. CDK12 regulates BNIP3 expression by interacting with FOXO3.
(A) The TIMER2.0 database indicates that among 10 candidate TFs in PCa, only RORA, FOXM1, FOXA1, HOXB13, FOXO3 and TCF12 are significantly associated with the expression of BNIP3. (B) The protein–protein interaction network of the 10 putative TFs with BNIP3 was analysed using STRING (Search Tool for the Retrieval of Interacting Genes/Proteins). (C) mRNA level of BNIP3 in PCa cells stably expressing control plasmid or shFOXO3 plasmid. (D) Western blotting analysis of input and co‐IP samples from HEK293T cells transfected with indicated plasmids. (E) Protein expression of FOXO3 protein in the cytoplasm and nucleus of C4‐2B cells transfected with shCDK2 plasmids.
**Figure S3**. Synergistic effect of CDK12 inhibition combined with enzalutamide in vitro and in vivo.
(A) Enzalutamide dose response curves in LNCaP cells with/without CDK12 kinase inhibitor THZ531 or CDK12 protein degrader BSJ‐4‐116 for 48 h. (B) THZ531 dose response curves in RM‐1 cells for 48 h. (C) Enzalutamide dose response curves in RM‐1 cells with/without THZ531 for 48 h. (D) Enzalutamide dose response curves in RM‐1 cells expressing shCtrl or shCDK12 plasmids. (E) Western blotting analysis from RM‐1 cells stably expressing shCtrl or shCdk12 plasmids. (F–H) Xenograft tumour growth curve and the collected tumours derived from RM‐1 cells expressing shCtrl or shCDK12 plasmids. Tumour volume at various time points of treatment is measured using a vernier calliper. The quantitation data represent means ± SD, *n* = 6. (I and J) HC analyses of Ki67, CDK12 and BNIP3 protein expression in RM‐1 cell‐derived xenograft tissues upon treatment with enzalutamide. Quantitation of Ki67+ cells represent means ± SD. Scale bar = 20 μm. (K) Protein expression of CDK12, and BNIP3 were determined by Western blotting.

**Table S1.** Information of chemical reagents.
**Table S2**. Sequences of shRNAs.
**Table S3**. Primers used for RT‐qPCR analysis.
**Table S4**. Information for primary antibodies.

## Data Availability

The data that support the findings of this study are available from the corresponding author upon reasonable request.
