# Supplementary material for: CDK12 Inactivation Attenuates Prostate Cancer Progression by Inhibiting BNIP3‐Mediated Mitophagy
Source: Cell Prolif. 2025 Jul 2;59(2):e70091. doi: 10.1111/cpr.70091 (PMC12877948; doi:10.1111/cpr.70091)
Supplement: Supplementary file 1 — Figure S1. Inhibition of CDK12 suppresses mitophagy. (A) CDK12 protein expression levels in various prostate cell lines. (B–E) PCa cells treated with THZ531 at gradually increasing doses were incubated with mitoSOX probe, and then flow cytometry analysis were performed to detect mitochondrial ROS levels. (F,G) Mitochondrial membrane potential was analysed by flow cytometry after incubation of PCa cells with the JC‐1 probe. (H–J) MitoTracker Red and MitoTracker Green staining (I), and quantification of mitochondrial mass (J) in PCa cells were measured by flow cytometry. The ratio of red to green fluorescence intensity, which indicated the number of heathy mitochondria, was quantified. (K) mtDNA copy numbers of PCa cells were measured by quantitative PCR assay after treated with CCCP and/or THZ531. (L and M) C4‐2B cells were treated with 10 μM CCCP and/or 100 nM THZ531 for 24 h. The protein levels of LC3 in mitochondria and the cytoplasm were measured by western blotting. The protein level of the LC3‐II form in mitochondria was quantified and normalised to VDAC1. Figure S2. CDK12 regulates BNIP3 expression by interacting with FOXO3. (A) The TIMER2.0 database indicates that among 10 candidate TFs in PCa, only RORA, FOXM1, FOXA1, HOXB13, FOXO3 and TCF12 are significantly associated with the expression of BNIP3. (B) The protein–protein interaction network of the 10 putative TFs with BNIP3 was analysed using STRING (Search Tool for the Retrieval of Interacting Genes/Proteins). (C) mRNA level of BNIP3 in PCa cells stably expressing control plasmid or shFOXO3 plasmid. (D) Western blotting analysis of input and co‐IP samples from HEK293T cells transfected with indicated plasmids. (E) Protein expression of FOXO3 protein in the cytoplasm and nucleus of C4‐2B cells transfected with shCDK2 plasmids. Figure S3. Synergistic effect of CDK12 inhibition combined with enzalutamide in vitro and in vivo. (A) Enzalutamide dose response curves in LNCaP cells with/without CDK12 kinase inhi [file CPR-59-e70091-s001.docx]

Supplementary Figures


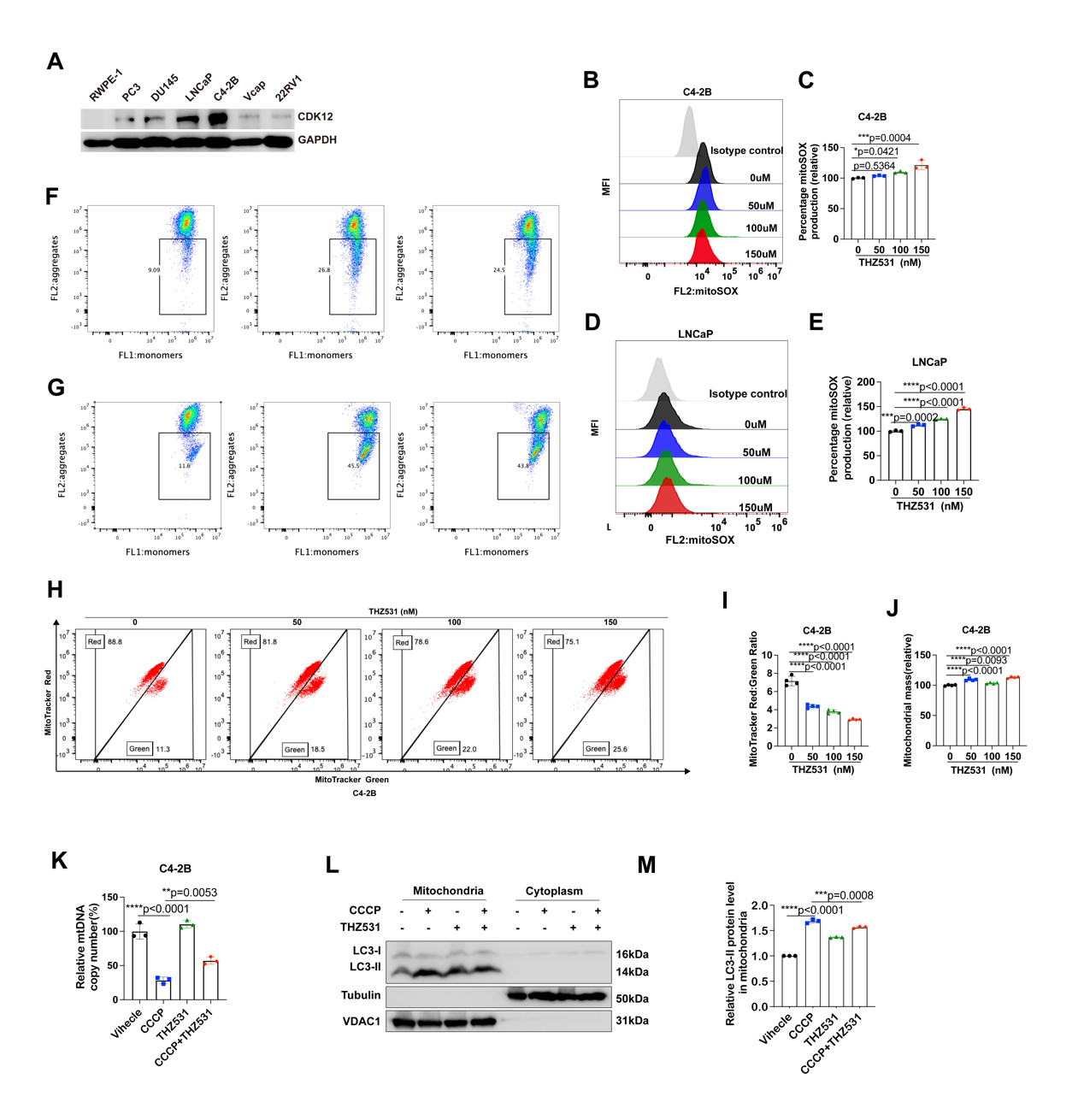


Figure S1. Inhibition of CDK12 suppresses mitophagy.

(A)​CDK12 protein expression levels in various prostate cell lines. (B-E)​ PCa cells treated with THZ531 at gradually increasing doses were incubated with mitoSOX probe, and then flow cytometry analysis were performed to detect mitochondrial ROS levels. (F,G) ​Mitochondrial membrane potential was analyzed by flow cytometry after incubation of PCa cells with the JC-1 probe. (H-J)​ MitoTracker Red and MitoTracker Green staining(I), and ​quantification of mitochondrial mass(J) in PCa cells were measured by flow cytometry. The ratio of red to green fluorescence intensity, which indicated the number of heathy mitochondria, was quantified. (K)​ mtDNA copy numbers of PCa cells were measured by ​quantitative PCR assay after treated ​with CCCP and/or THZ531. (L,M) ​C4-2B cells were treated with 10 μM CCCP ​and/or 100 nM THZ531 for 24 h. The protein levels of LC3 in mitochondria and the cytoplasm were measured by western blotting. The protein level of the LC3-II form in mitochondria was quantified and normalized to VDAC1.


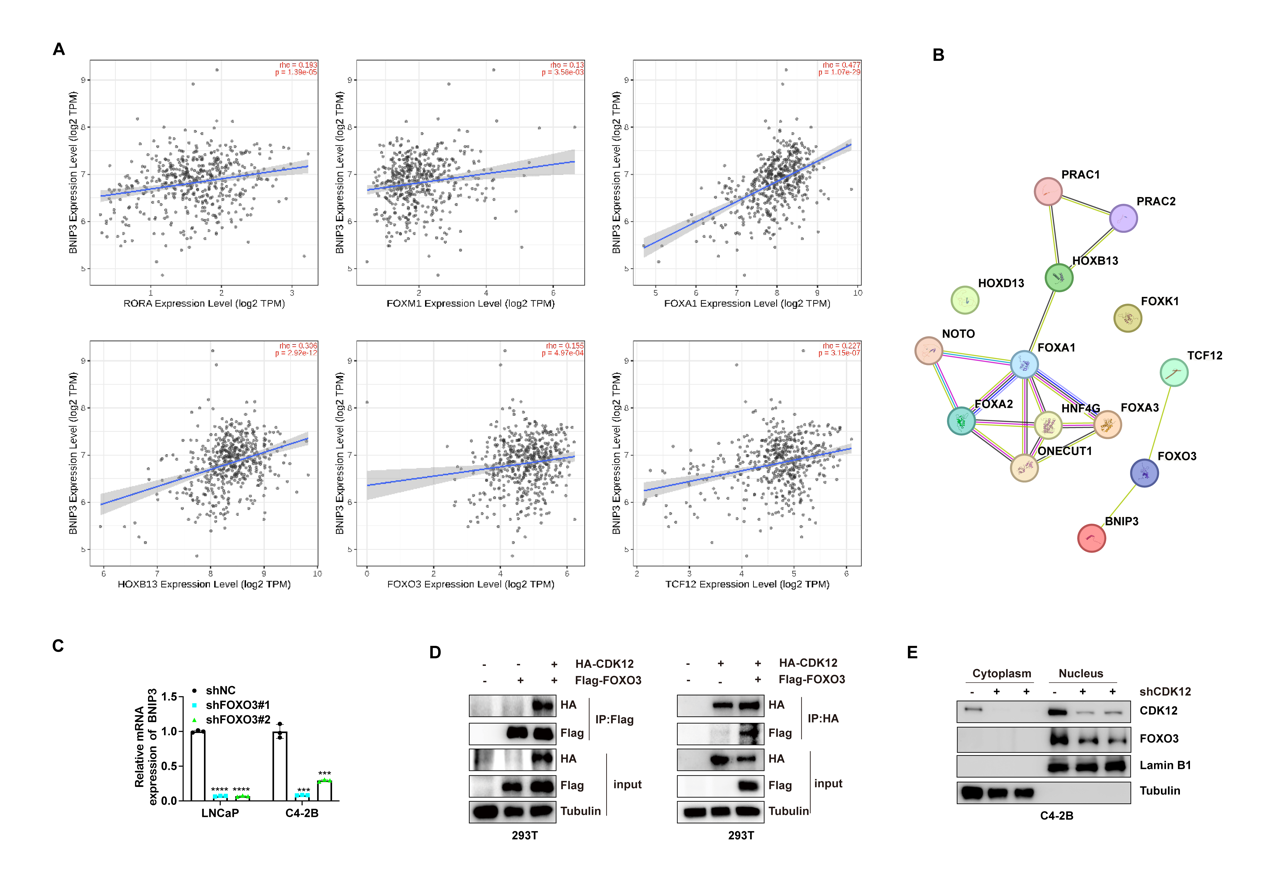


​​Figure S2. ​CDK12 regulates BNIP3 ​expression by interacting with FOXO3.

(A) ​The TIMER2.0 database indicates that among 10 candidate TFs in PCa, only RORA, FOXM1, ​FOXA1, HOXB13, FOXO3, and TCF12 are significantly associated with the expression of BNIP3. ​(B) The protein-protein interaction network of the 10 putative TFs with BNIP3 was analyzed using STRING (Search Tool for the Retrieval of Interacting Genes/Proteins). ​(C) ​mRNA level ​of BNIP3 in PCa cells stably expressing control plasmid or shFOXO3 plasmid.​ ​(D) Western blotting analysis of input and co-IP samples from HEK293T cells transfected with indicated plasmids. ​(E) ​Protein expression of FOXO3 protein in the cytoplasm and nucleus of C4-2B cells ​transfected with shCDK2 plasmids.


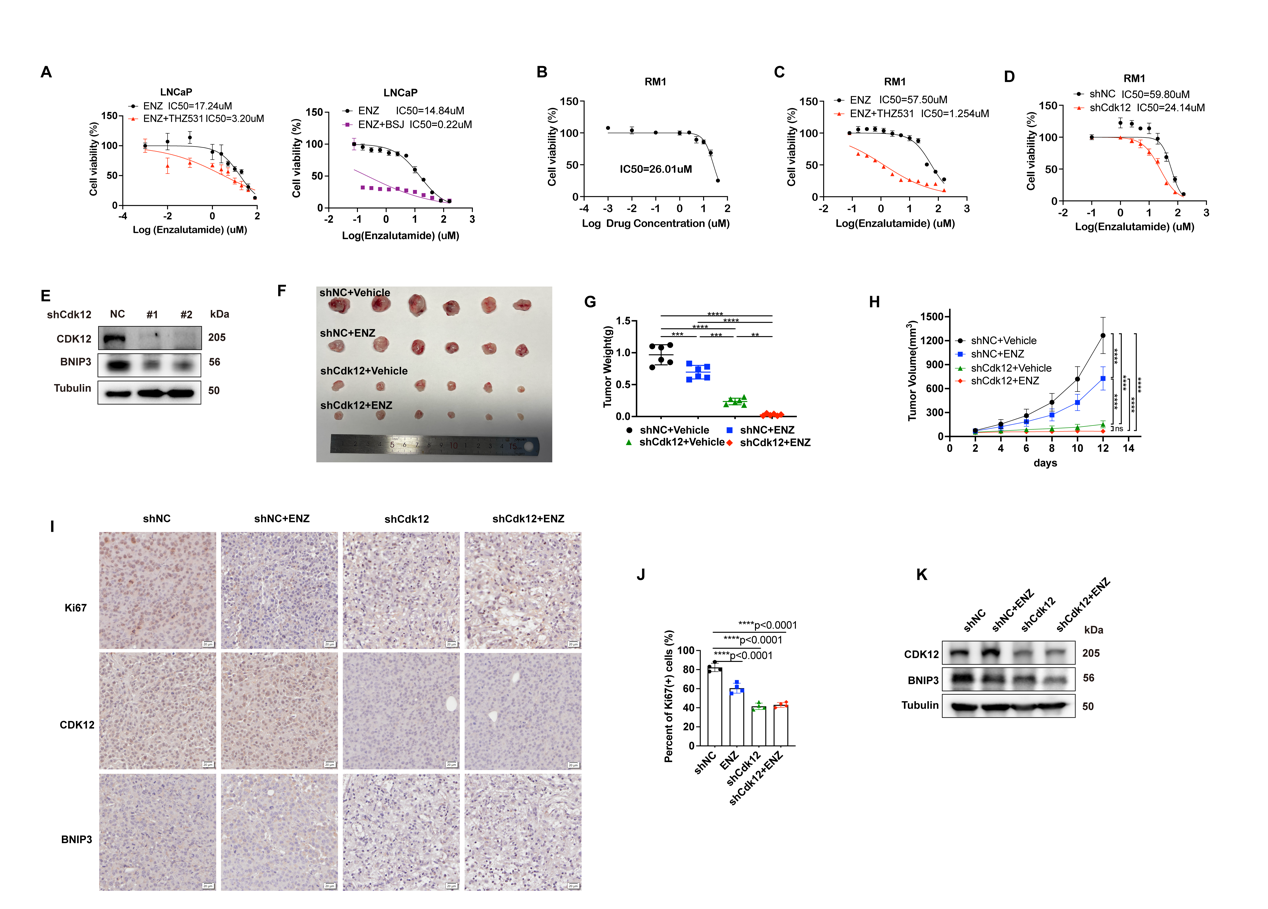


​​Figure S3. ​Synergistic effect of CDK12 inhibition combined with enzalutamide in vitro and in vivo.

​(A) Enzalutamide dose response curves in LNCaP cells with/without CDK12 kinase inhibitor THZ531 or CDK12 protein degrader BSJ-4-116 for 48h. (B) THZ531 ​dose response curves in RM-1 cells​ for 48h. (C)​ Enzalutamide dose response curves in RM-1 cells with/without THZ531 for 48h. ​(D) ​Enzalutamide dose response curves in RM-1 cells expressing shCtrl or shCDK12 ​plasmids. (E) ​Western blotting analysis from ​RM-1 cells stably expressing shCtrl or shCdk12 ​plasmids. (F-H) ​Xenograft tumor growth curve and the collected tumors derived from RM-1 cells ​expressing shCtrl or shCDK12 plasmids. Tumor volume at various time points of treatment is measured using a vernier caliper. The quantitation data represent means ± SD, n = 6. (I,J)​ HC analyses of Ki67, CDK12 and BNIP3 protein expression in RM-1 cell-derived xenograft tissues upon treatment with enzalutamide. Quantitation of Ki67+ cells represent means ± SD. Scale bar = 20 µm. (K) ​Protein expression of CDK12, and BNIP3 were determined by Western blotting.
