# Supplementary material for: CDK12 Inactivation Attenuates Prostate Cancer Progression by Inhibiting BNIP3‐Mediated Mitophagy
Source: Cell Prolif. 2025 Jul 2;59(2):e70091. doi: 10.1111/cpr.70091 (PMC12877948; doi:10.1111/cpr.70091)
Supplement: Supplementary file 2 — Table S1. Information of chemical reagents. Table S2. Sequences of shRNAs. Table S3. Primers used for RT‐qPCR analysis. Table S4. Information for primary antibodies. [file CPR-59-e70091-s002.docx]

**Supplementary Table 1. Information of chemical reagents.**

| **Chemicals** | **Vendors** | **Cat#** |
| --- | --- | --- |
| THZ531 | MCE | HY-103618 |
| Enzalutamide | MCE | HY-70002 |
| Mdivi-1 | MCE | HY-15886 |
| CCCP | MCE | HY100941 |
| MitoSOX Red | MCE | HY-D1055 |
| Chloroquine | MCE | HY-17589A |
| MitoTracker Green FM | MeilunBio | MB6044 |
| MitoTracker Red CMXRos | MeilunBio | MB6046 |

**Supplementary Table 2. Sequences of shRNAs.**

| **Plasmids** | **Sequences** |
| --- | --- |
| shNS | CCGGCAACAAGATGAAGAGCACCAACTCGAG  TTGGTGCTCTTCATCTTGTTGTTTTT |
| shCDK12#1 | CCGGGCACTGAAAGAGGAGATTGTTCTCGAGAACAATCTCCTCT  TTCAGTGCTTTTT |
| shCDK12#2 | CCGGGCGTTCCCGGGACTTACTAAACTCGAGTTTAGTAAGTCCC  GGGAACGCTTTTTG |
| shCdk12#1 | CCAGAAGTAGAAGTCCTGCATTTCAAGAGAATGCAGGACTTCTACTTCTGG |
| shCdk12#2 | GCACCGAAAGAGGTGATTGTTCTCGAGAACAATCACCTCTTTCGGTGC |
| shFOXO3#1 | cgctcgaagtggagctggaccTTCAAGAGAggtccagctccacttcgagcg |
| shFOXO3#2 | cgaggaggaggacgatgaagaTTCAAGAGAtcttcatcgtcctcctcctcg |

**Supplementary Table 3. Primers used for RT-qPCR analysis**

| **Genes** | **Primers** | **Sequences** |
| --- | --- | --- |
| *BNIP3* | Forward | CCTCAGCATGAGGAACACGA |
|  | Reverse | AAAAGGTGCTGGTGGAGGTT |
| *FOXO3* | Forward | TGCGTGCCCTACTTCAAGGATAA |
|  | Reverse | ACAGGTTGTGCCGGATGGA |
| *CDK12* | Forward | CCCTGGTGAAGAACAGGACC |
|  | Reverse | GGTGTAACGCTAAGGGGACC |
| *ACTB* | Forward | GATCATTGCTCCTCCTGAGC |
|  | Reverse | GGGCCGGACTCGTCATA |

**Supplementary Table 4. Information for primary antibodies**

| **Antibodies** | **Vendors** | **Cat#** |
| --- | --- | --- |
| CDK12 | Cell Signaling Technology | 11973 |
| CDK12 | ProteinTech | 26816-1-AP |
| FOXO3 | ProteinTech | 66428–1-Ig |
| LC3 | ProteinTech | 14600–1-AP |
| Lamin B1 | ProteinTech | 66095-1-Ig |
| GAPDH | ProteinTech | 60004–1-lg |
| PINK1 | ProteinTech | 23274-1-AP |
| PRKN | ProteinTech | 14060-1-AP |
| Beta Tubulin | ProteinTech | 10094-1-AP |
| p-FOXO3A(S253) | ABclonal | AP0684 |
| Bcl2-L-13 | HUABIO Technology | HA500329 |
| FUNDC1 | HUABIO Technology | HA721530 |
| BNI3L | HUABIO Technology | HA722056 |
| TOMM20 | Abcam | ab186735 |
| Goat Anti-Mouse IgG | Abbkine | A21010 |
| Goat Anti-Rabbit IgG | Abbkine | A21020 |
